# Barriers and Facilitators to Remote Tai Chi for Knee Osteoarthritis in Large Healthcare Systems: A Qualitative Study

**DOI:** 10.1093/geroni/igaf122.2125

**Published:** 2025-12-31

**Authors:** Eric Roseen, Lance Laird, Rocky Reichman, Ludovic Trinquart, Brian Mittman, Helen Lavretsky, Robert Saper, Chenchen Wang

**Affiliations:** Boston University, Boston, Massachusetts, United States; Boston University, Boston, Massachusetts, United States; Boston University, Boston, Massachusetts, United States; Tufts University, Medford, Massachusetts, United States; Kaiser Permanente Southern California, Pasedena, California, United States; University of California, Los Angeles Healthcare, Los Angeles, California, United States; Cleveland Clinic, Cleveland, Ohio, United States; Tufts University, Medford, Massachusetts, United States

## Abstract

While clinical practice guidelines from the American College of Rheumatology strongly recommend Tai Chi for treating knee osteoarthritis (OA), adoption of Tai Chi for Knee OA in healthcare systems is low. Our aim is to identify multi-level barriers and facilitators to embed a remote Tai Chi intervention for knee OA in four large healthcare systems. We conducted 30- to 60-minute interviews among individuals from four healthcare systems in California, Ohio, Florida, and Massachusetts from September 2023 to June 2024. Recorded semi-structured interviews were transcribed verbatim. Participants were asked about factors influencing use of in-person or remotely delivered Tai Chi. Major themes were identified using thematic content analysis and an a priori codebook based on the Consolidated Framework for Implementation Research. We interviewed 59 participants (Median age=58 [range=36-85] years; 54% women; 42% White) across four large healthcare systems (13 patients, 16 primary care providers, 12 Tai Chi instructors, 18 health system leaders). Logistical challenges (e.g., transportation, scheduling, technology access) and lack of insurance coverage were considered main barriers. PCPs were also unsure how to describe Tai Chi and did not have a mechanism for making referrals or communicating with Tai Chi instructors via the electronic health record. These results have informed recruitment efforts to address known barriers in an ongoing multi-site embedded pragmatic trial of remote Tai Chi for 480 patients with knee osteoarthritis. However, long-term adoption may also rely on addressing policy barriers such as insurance coverage for tai chi or credentialling of tai chi instructors in large healthcare systems.

